# Image-Based High-Throughput Detection and Phenotype Evaluation Method for Multiple Lettuce Varieties

**DOI:** 10.3389/fpls.2020.563386

**Published:** 2020-10-06

**Authors:** Jianjun Du, Xianju Lu, Jiangchuan Fan, Yajuan Qin, Xiaozeng Yang, Xinyu Guo

**Affiliations:** ^1^Beijing Academy of Agriculture and Forestry Sciences, Beijing, China; ^2^Beijing Key Lab of Digital Plant, Beijing Research Center for Information Technology in Agriculture, Beijing, China; ^3^Beijing Key Laboratory of Agricultural Genetic Resources and Biotechnology, Beijing Agro-Biotechnology Research Center, Beijing, China

**Keywords:** high throughput phenotyping, lettuce, object detection, semantic segmentation, static trait, dynamic trait, growth rate

## Abstract

The yield and quality of fresh lettuce can be determined from the growth rate and color of individual plants. Manual assessment and phenotyping for hundreds of varieties of lettuce is very time consuming and labor intensive. In this study, we utilized a “Sensor-to-Plant” greenhouse phenotyping platform to periodically capture top-view images of lettuce, and datasets of over 2000 plants from 500 lettuce varieties were thus captured at eight time points during vegetative growth. Here, we present a novel object detection–semantic segmentation–phenotyping method based on convolutional neural networks (CNNs) to conduct non-invasive and high-throughput phenotyping of the growth and development status of multiple lettuce varieties. Multistage CNN models for object detection and semantic segmentation were integrated to bridge the gap between image capture and plant phenotyping. An object detection model was used to detect and identify each pot from the sequence of images with 99.82% accuracy, semantic segmentation model was utilized to segment and identify each lettuce plant with a 97.65% F1 score, and a phenotyping pipeline was utilized to extract a total of 15 static traits (related to geometry and color) of each lettuce plant. Furthermore, the dynamic traits (growth and accumulation rates) were calculated based on the changing curves of static traits at eight growth points. The correlation and descriptive ability of these static and dynamic traits were carefully evaluated for the interpretability of traits related to digital biomass and quality of lettuce, and the observed accumulation rates of static straits more accurately reflected the growth status of lettuce plants. Finally, we validated the application of image-based high-throughput phenotyping through geometric measurement and color grading for a wide range of lettuce varieties. The proposed method can be extended to crops such as maize, wheat, and soybean as a non-invasive means of phenotype evaluation and identification.

## Introduction

Lettuce is an economically important vegetable crop widely cultivated in the world, with the highest outputs in the United States, Europe, and China (Adhikari et al., 2019). Rich in vitamins, carotenoids, antioxidants, and other phytonutrients (Humphries and Khachik, 2003), lettuce is the most consumed leafy vegetable in salads in Europe and the United States and also one of the most commonly used vegetables in Chinese hotpot cuisine. As a leafy vegetable of great economic value, lettuce can be harvested at maturity or in early development (Simko et al., 2016). Lettuce leaves are the main organs of biomass accumulation, and lettuce leaves can be harvested many times during vegetative growth. Therefore, rapid growth of lettuce leaves is beneficial to ensuring the production of leaves with the same shape, color, and taste (Grahn et al., 2015). In addition, environmental factors have important effects on gene expression, protein level, chlorophyll content, photosynthesis, and metabolites of lettuce. Through the appropriate use of culture substrates in a greenhouse, the environmental differences among lettuce varieties can be eliminated to a large extent, simplifying the evaluation of the growth and development status of lettuce plant and clarifying the relationship between the phenotype and genotype.

Image capture is a low-cost and efficient way to assess plant growth status, and industrial cameras have become the basis of almost all high-throughput phenotyping platforms. Lettuce breeders have used optical sensors to evaluate the vegetative growth of lettuce plants and to conduct genetic studies on lettuce by, for example, identifying and mapping the locus controlling light green leaf color (qLG4) in lettuce (Simko et al., 2016). In addition, heat-sensitive and heat-resistant lettuce can also be screened by measuring the leaf and root morphology, and the effect on the leaf and root morphology of RILs population can be quantified to establish a basis for breeding new varieties of lettuce (Choong et al., 2013). Conditions, such as temperature or soil salinity, are critical to the growth of lettuce, which is very sensitive to increases in soil or water salinity (NaCl/CaCl_2_). Increased salt concentrations inhibit the growth of lettuce, which is especially sensitive at the early stage of development (Xu and Mou, 2015). Studying the dynamic response of lettuce to different abiotic stresses is an important way to evaluate the resistance, e.g., salt tolerance, of a large number of germplasm resources (Zhou et al., 2018). Existing imaging platforms (e.g., PlantScreen^TM^, developed by Photon Systems Instruments) are also used for dynamic growth analysis of lettuce plants (Sorrentino et al., 2020), but usually only a few types of lettuce can be processed. There remains a lack of research quantifying and evaluating the dynamic growth of large-scale lettuce cultivation using high-throughput phenotyping platforms.

To continuously, stably, and consistently evaluate the growth status of a wide range of lettuce varieties, high-throughput image acquisition is a particularly effective tool. Two modes, i.e., “Plant to Sensor” (Rajendran et al., 2009) and “Sensor to Plant” (Kjaer and Ottosen, 2015), are usually adopted to collect high-throughput data from plants. The former requires complex mechanical structures to transfer plants to a fixed imaging room for data acquisition. Its advantage is that plant images can be captured from different angles; however, the cost of such platforms is very high, and the related work procedures are more complex. The latter system only needs to control the movement of a sensor to specified positions to perform data collection, and it usually only collects images from the overhead views of plant; thus, it is relatively simple and efficient. The present work utilized the “Sensor to Plant” approach to obtaining large quantities of top-view images in a greenhouse environment. Thousands of lettuce images can thus be obtained in a short period of time, and this approach guarantees time-effectiveness for 500 lettuce varieties. The growth and color status of lettuce canopies and leaves can be reflected in top-view images, which can therefore be used as a reliable data source for subsequent phenotypic analyses of lettuce.

Based on high-throughput image acquisition, efficient and high-precision image analysis becomes the key to transforming image data into comprehensive plant traits. For the traits considered by crop breeders, image-based phenotyping pipelines are usually customized based on new or improved image processing methods. In this context, convolutional neural networks (CNNs) are one of the basic components of image-based phenotyping methodologies. CNNs originated in the 1980s (Fukushima, 1980) and became areas of intense active research after Lenet-5 (LeCun et al., 1998) was proposed, which directly promoted the recovery of neural networks and the rise of deep learning through the emergence of AlexNet in 2012 (Krizhevsky et al., 2012). Owing to the leading performance advantages of CNNs in image classification, object detection, semantics, and instance segmentation, image-based phenotyping deeply integrated with CNNs has been widely used to extract and evaluate crop traits for genetics, genomics, breeding, and agricultural production studies (Lu et al., 2017; Taghavi Namin et al., 2018; Ubbens et al., 2018; Uzal et al., 2018).

The objective of this work is the establishment of a data analysis pipeline for high-throughput detection and phenotyping of multiple lettuce varieties. A high-throughput phenotyping platform was utilized to periodically acquire top-view images of lettuce plants. We then built an image analysis pipeline aimed at assessing lettuce growth, which used integrated object detection and semantic segmentation models based on CNN, to extract information from each pot and plant. Finally, we assessed the static and dynamic traits of multiple lettuce varieties, and validated this application of image-based high-throughput phenotyping in geometric measurement and color grading for a broad range of lettuce varieties.

## Materials and Methods

### Data Acquisition

**FIGURE 1:**
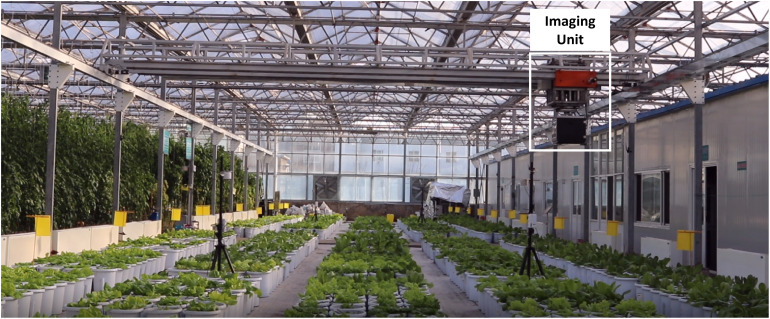
High-throughput phenotyping platform for greenhouses. The imaging unit is driven along a route with planned according to *x* and *y* coordinates, and top-view images of lettuce plants are captured according to a timer or its position.

The experimental data acquisition system was built in the greenhouse of the Beijing Academy of Agricultural and Forestry Sciences, utilizing a planar scanning motion mechanism. The imaging unit was able to move automatically above greenhouse regions according to a planned route (Figure 1). The imaging unit was 2.3 m above the greenhouse floor. A mounted industrial camera (Point Gray, Sony ICX808, 1/1.8”, 3.1 m, Global shutter, 18 FPS at 2016 × 2016 pixels) was used to continuously and systematically obtain top-view images of plants. The time interval of data collection was set according to the movement speed of the imaging unit. The total time of a complete data collection process was 38 min, during which time 2280 images could be obtained. The greenhouse was equipped with an automated irrigation system, which could supply water according to the water status of each plant to ensure the normal growth of plants. This experiment began in mid-December 2019, when lettuce seeds were first sown in a square basin (68 mm × 68 mm; depth, 95 mm) with a 1:1 soil–sand mixture, and seedlings were transplanted into pots (diameter, 32 cm; height, 34 cm) in the greenhouse after 30 days.

Into each of 2000 pots, one or two lettuce plants were transplanted, and one plant was retained after 3 days. Plant data were collected at 16:00 p.m. every other day. At that time, the photo-period and photosynthesis rate in the greenhouse was fairly uniform across all lettuce varieties (Simko et al., 2016). In this work, image datasets at eight key time points of lettuce growth were used to evaluate the static and dynamic traits of lettuce plants.

### Data Analysis Pipeline

We designed the data analysis pipeline to automatically process the image datasets. For the top-view images of lettuce plants at each time point, data cleaning and calibration were performed to reduce the data storage and analysis demands. In a single data collection session, thousands of images could be collected, but a few images (redundant images) were not included in the comprehensive images of lettuce cultivation regions. Thus, data cleaning was performed to remove the redundant images of the same pot, according to the image acquisition time and location, prior to the subsequent image analysis. The calibration step converted the pixel size of the collected images by detecting the fixed calibration object. The pixel size of the collected image in this work was 1.067 mm/pixels. In order to ensure the matched accuracy of the same plant among different growth points, we used pot detection instead of plant detection. Therefore, after cleaning, the image sequences were successively processed by the pot detection model. It is worth noting that each pot might appear in multiple images at the same time in the image sequence, but the relative positions of each pot in the adjacent images were different. The detection results were used to establish the mapping relationship between pots and the sequence of images and further used to identify the most appropriate top-view image of each pot.

Pot detection for many of the images was a highly redundant operation. We trained an efficient pot detection model based on a CNN to extract pots from the image sequence, and each pot was associated with the size and local coordinates of a certain image. The pot closest to the image center among the images captured had the least imaging position distortion, and this image could thus be regarded as the most suitable top-view image of this pot. Therefore, according to the local position of the pot in the image and image acquisition location, the spatial position of each pot could be determined. Since each pot corresponded to a lettuce plant, we could also determine the spatial position of the lettuce plant according to its pot position. In this way, the pot could be matched to a position, a variety, and a plant. By the late stages of growth, plant leaves would usually grow beyond the perimeter of the pot, possibly causing interference effects among plants. In order to uniformly quantify and evaluate the dynamic growth process of plants over 34 days, we evaluated static and dynamic traits of lettuce only within the pot region.

We trained a semantic segmentation model to extract the lettuce plants from pot images, and the segmented results were then fed into the phenotyping analysis pipeline to calculate the static traits describing, for example, the geometry and color of lettuce. Finally, the image datasets collected at different time points were used to calculate dynamic traits, such as the growth rate and cumulative growth rate of each static trait (Figure 1).

The above data analysis pipeline was implemented in Python under the Windows 10 operating system. The computer used had the following specifications: Intel(R) Core^TM^ i7-5930k CPU@3.50GHz, 128G RAM, two 8GB NVIDIA GeForce GTX-1080 Ti graphics card, 2TB hard disk. This computer was mainly used to train CNN models for object detection and semantic segmentation (within the TensorFlow framework), as well as for automatic analysis of large quantities of image datasets.

### Detection Model

**FIGURE 2:**
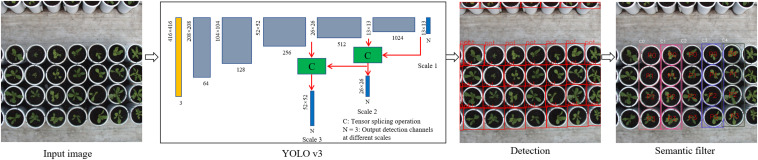
Pot detection model and post-processing. Input images were scaled to 416 × 416 pixels and fed into the YOLO v3 model, resulting in a series of predicted boxes. Semantic filtering was performed to extract the valid boxes and assign the local variety and plant indices. C indicates tensor splicing operation, *N* = 3 represents the output detection channels at different scales, ‘pot’ represents the detected pot, ‘C-’ is the variety index, and ‘P-’ represents the plant index of the individual variety.

Accurate and efficient pot detection is the key to accurately locating and extracting lettuce plants and a precondition for quantifying plant traits. The YOLOv3 algorithm model (Redmon and Farhadi, 2017) was implemented to perform the end-to-end detection of pots from the top-view image sequence, as shown in Figure 2. The network structure was used to train the pot detection model based on a sufficient number of annotated pot datasets. This detection model determined the positions of pots by using regression; thus, the corresponding prediction boxes of each pot could be obtained by evaluating each image once. The DarkNet-53 network was used to extract features, and the residual structure of ResNet (He et al., 2016) was introduced to control the gradient spread. The network consists of 53 consecutive 3 × 3 and 1 × 1 convolution layers, with the 3 × 3 convolution responsible for increasing the feature graph channel and the 1 × 1 convolutional layer responsible for compressing feature representation after the 3 × 3 convolution. Five 3 × 3 convolutional layers with a step length of 2 were used at intervals to reduce the output feature image to 1/32 the size of the input image. One important characteristic of this structure is it merges the multiscale features to obtain more discriminating feature description. Consequently, it can be independently detected from three feature graphs with 13 × 13, 26 × 26, and 52 × 52 scales so as to meet the detection needs for objects of different sizes. The network allocated three anchor boxes for each scale, corresponding to a total of nine anchor boxes. In this paper, a *k*-means clustering algorithm was used to re-cluster all annotated boxes in the training dataset, and the clustered box list for this dataset was used as a model training.

The pot detection process first involved the size of collected images being scaled to 416 × 416 pixels, and this data was then fed into a pot detection model. The end-to-end detection resulted in a series of predicted boxes, which contained confidence values and relative coordinates. Then, object boxes with confidence values lower than the threshold value (0.9) were removed by non-maximum suppression (NMS), and each remaining detection box then corresponded to a predicted pot. Further, we defined a region of interest (ROI) in each image to judge whether each predicted box was valid or not, and only those boxes that were completely contained within the ROI were retained. According to the relative position and global position of the pot in each image, the corresponding variety and plant indices of the pot were attached to the predicted box. Based on the above operation, we could extract and identify pots from the image sequence and process them using the semantic segmentation described in the next section.

### Semantic Segmentation

Under greenhouse environments, lettuce planting density is high, but growth rates differ among varieties. In the later stage of growth, lettuce leaves gradually grow beyond the perimeters of their pots and interfere with neighboring plants. From the top-view, the early growth range of lettuce is confined to within each pot, and there is no interference among neighboring plants. However, in the later stages of vegetative growth, most lettuce varieties will gradually grow beyond the pot area. In this paper, only plants within the pot regions were semantically segmented. The pot region images of each variety and each plant were extracted from the image sequences data used for image analysis.

**FIGURE 3:**
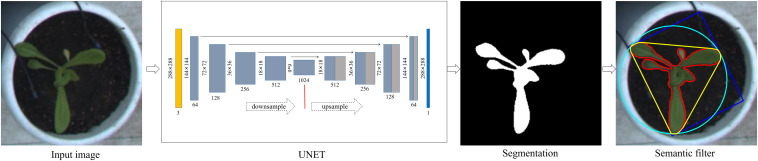
Plant semantic segmentation and post-processing. Input images were scaled to 218 × 218 pixels and fed into a UNET model, resulting in a binary image. Semantic filtering was performed to check the valid regions for subsequent feature extraction.

Figure 3 shows the semantic segmentation of plants and its post-processing process. We developed a semantic segmentation model of lettuce by using the U-Net network structure (Ronneberger et al., 2015). The network adopts the classical encoding/decoding structure, which mainly includes encoder, decoder, and jump connection components. Among them, the encoder, which is mainly composed of 3 × 3 convolution and 2 × 2 maximum pooling layers, is used to extract features from images at different scales. The activation function is ReLU, and the feature channel is doubled after each encoding. The decoder utilizes deconvolution to gradually halve the number of channels and splice the deconvolution result with the feature graph on the corresponding encoding path. After decoding, the output feature graph is restored to the size of the input image. The jump connection establishes the channel connection between the encoder and decoder features, which can reduce the information loss in feature extraction and improve the accuracy of location and segmentation.

The semantic segmentation process was as follows: pot images that were exported from the detection model were adjusted to 288 × 288 pixels and imported into the UNET network. The model output followed a probability distribution ranging between 0 and 1, and based on this output, we used a fixed value as the threshold to convert the probability graph into a binary image. We then detected the maximum connected region in the binary image and regarded it as the main structure of each lettuce plant for the subsequent feature extraction.

### Feature Extraction

To quantify the dynamic growth processes of lettuce plants during vegetative growth, we extracted data from each pot image and segmented plants from the pot images. This provided the basis for analyzing and evaluating lettuce traits. The top-view images of lettuce plants contain structure, shape, and color information of the plant canopy and leaves, which is an intuitive reflection of growth status and variation. We focused on the static traits (SI) of lettuce across growth points, as well as the dynamic traits corresponding to static traits during a period of growth, including growth rate (GR) and accumulation rate (AC), as shown in Table 1. In this work, the static traits refer to the geometric and color indices of lettuce plants at certain time points, and the growth rates and cumulative traits reflect changes in the static traits during a particular growth period. Therefore, static and dynamic traits are closely related to data acquisition times and time periods. The growth rate describes the change rate of various static traits within a certain time interval, and the cumulative traits corresponding to the static traits are the average daily change rates within a certain time interval, as follows:

**TABLE 1:** Lettuce features extracted from top-view images.

	**Static Index (SI)**	**Growth rate index (GR)**	**Accumulated curve Index (AC)**	**Description**	**Unit or value range**
**Geometric Descriptors**	PA	GRPA	ACPA	Projected area	cm^2^
	PP	GRPP	ACPP	Projected perimeter	cm
	CA	GRCA	ACCA	Convex hull area	cm^2^
	CP	GRCP	ACCP	Convex hull perimeter	cm
	AXL	GRAXL	ACAXL	Long axis length of OBB (OBB, the oriented bounding box)	cm
	AXS	GRAXS	ACAXS	Short axis length of OBB	cm
	PAR	GRPAR	ACPAR	PA/CCA (CCA, Circumscribed area)	/
	CAR	GRCAR	ACCAR	PA/CA	/
**Color Descriptors**	R	GRR	ACR	Mean red	[0,255]
	G	GRG	ACG	Mean Green	[0,255]
	B	GRB	ACB	Mean Blue	[0,255]
	H	GRH	ACH	Mean Hue	[0,255]
	S	GRS	ACS	Mean Saturation	[0,255]
	V	GRV	ACV	Mean Value	[0,255]
	ExG	GRExG	ACExG	Normalize 2G-R-B (Woebbecke et al., 1995)	[0,1]

(1) $$\left\{ \begin{array}{c} G\,R_{S\,I}=\frac{S\,I_{t\,2}-S\,I_{t\,1}}{t\,2-t\,1}\\ A\,C_{S\,I}=\frac{\sum_{t\,1}^{t\,2}S\,I}{t\,2-t\,1} \end{array}\right..$$

Here, SI represents the static indices in Table 1; *t1* and *t2* respectively represent the start and end times of data acquisition, respectively, where the unit is day; *GR*_*SI*_ and *AC*_*SI*_ represent the growth rate and cumulative traits of static traits (SI), respectively.

## Results

### Training the Detection Model

**FIGURE 4:**
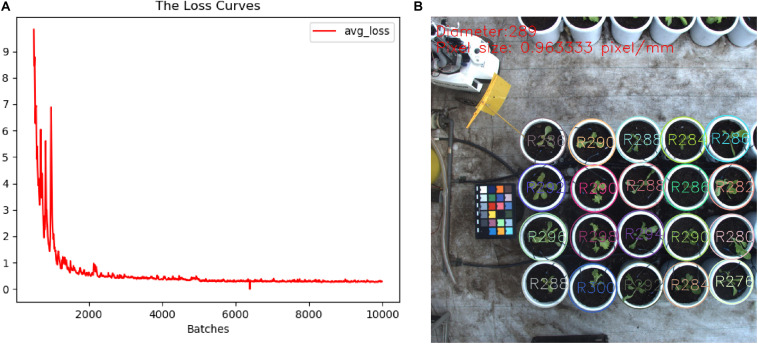
Training detection model. **(A)** Loss curve. **(B)** Detected pots and their sizes in the input image.

To obtain a high-accuracy detection model, we collected different types of images from several imaging devices to establish pot annotation datasets. These images were captured by industrial cameras, digital cameras (Canon77D), and the Kinect2 sensor, which yielded image of 2016 × 2016, 6000 × 4000, and 1920 × 1080 pixels, respectively. A total of 236 images covered different lighting conditions and imaging heights. Multiple types and sizes of images are helpful to improve the robustness and adaptability of detection models. It should be noted that these images were mainly collected within 30 days after initial lettuce cultivation. We only focused on a single category of object (i.e., pots) in the images, so we thus developed a simple interactive box-selection tool to extract pots from each image and then stored data from each pot in the a corresponding TXT file as five parameters, i.e., category, relative coordinates (i.e., *x*, *y*), and size (i.e., width, height). As a result, a total of 7,204 pots of different sizes were labeled manually from these images. Then, the source images and their annotation results were rotated at the same time to expand the number of images three-fold. Finally, 2/3 of images were randomly selected to construct the training dataset, and the remaining images were used as the test dataset. In order to reduce the risk of over-fitting, image operations, such as random scaling and clipping, image exposure, and saturation, were used when the model was loaded into the training model (Barth et al., 2019). A weight model (darknet53) obtained from ImageNet data was used to initialize model training. The basic parameters were set as follows: batch size was 64; the optimizer was Adam; the initial learning rate was 0.001, which was reduced to 0.1 times that of the previous learning rate at 5000 and 8000 iterations, respectively. After 10,000 iterations of model training, the model training loss curve shown in Figure 4A was generated. Through the training and testing, the mean average precision of the average accuracy (mAP@0.50) of this model reached 99.82%, and the recognition accuracy, recall rate, and average intersection over union (IOU) were 96.8, 90.64, and 87.13%, respectively. Figure 4B shows the detection and post-processing results of the images with sizes of 2016 × 2016, and the model detection efficiency reached 0.2 s. The pot diameters detected from the same image were not completely consistent, which is mainly related to the position distortion and annotation tolerance for pots in the training set. The average diameter and pixel size of the pots had an error of less than 3%.

### Training Semantic Segmentation Model

To train a high-robustness semantic segmentation model, we collected 5913 pot images from different growth points and lettuce varieties and established a pot dataset. We developed an interactive contour-editing tool to ensure that the lettuce (if any) in the pot image would be accurately extracted, and we then stored these contours in the corresponding mask image. Thus, pot images and their corresponding mask images together comprised an image dataset for semantic segmentation. Further, this dataset was divided into training, verification, and test datasets, containing 3548, 1183, and 1182, images respectively. The parameter configuration of the semantic segmentation model was as follows: the activation function was SIGMOID; batch size was 12, to maximize GPU storage; epochs numbered 100; the optimizer was Adam; the learning rate (LR) was set as 5×10^−5^. The loss function of the model combined DICE and Binary Focal Loss (Lin et al., 2020), and IOU was used to evaluate the segmentation performance. The Inceptionresnetv2 network (Szegedy et al., 2017) was used to extract features.

**FIGURE 5:**
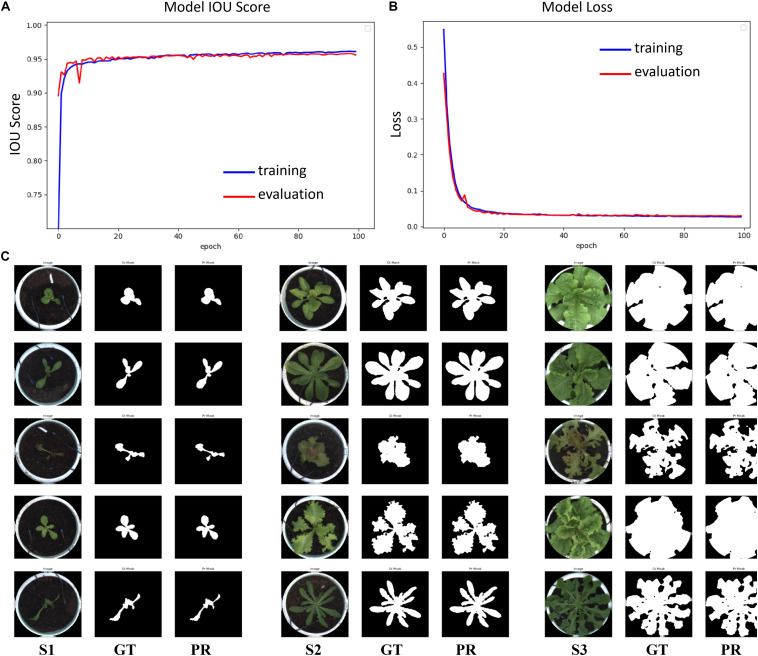
Evaluation results of the semantic segmentation model. **(A)** IOU score, **(B)** Loss curve, **(C)** Segmentation results of lettuce plants in the test dataset at different growth points (S1, S2, and S3 respectively correspond to three growth points; GT refers to the manual annotation image in the test dataset; PR refers to prediction results of semantic segmentation).

Image augmentation techniques, such as random flipping, rotation, scaling, perspective, blurring, sharpening, contrast, and brightness adjustment, were also used to generate the dataset (Buslaev et al., 2020) to improve the data generalization. The model was trained 100 times, and the IOU score and loss curves of the model are shown in Figures 5A,B. The loss rate, IOU score, and F1 score of the model on the training set are shown in Table 2. The loss rate was about 3%, the average accuracy (IOU, mAP) was over 95%, and the average F1 score (the harmonic average of precision and recall) was over 97%. The test dataset included 1,182 images of different lettuce varieties at different growth points, with a total processing time of about 76 s, or approximately 64 ms per plant. Three types of original images at different growth points (S1, S2, and S3), labeled images (GT), and prediction images (PR) were utilized to evaluate the segmentation accuracy, as shown in Figure 5C. The visual comparison between the artificial annotation and the model prediction images shows that the main plant structures could be extracted, and the main differences occurred owing to the presence of slender stems, leaf edges, and internal pores.

**TABLE 2:** Performance of semantic segmentation model on training, verification, and test datasets.

**Dataset type**	**Number**	**Loss**	**Average IOU**	**Average F1**
**Training set**	3548	0.0269	0.9610	0.9800
**Validation set**	1183	0.0304	0.9557	0.9768
**Test set**	1182	0.0311	0.9549	0.9765

### Evaluating Static Traits

Different lettuce varieties have different geometric and color traits. In this paper, 2036 images of pots (including 36 pots from other experiments) were obtained on the same day to analyze the correlation between 15 geometry and color traits of lettuce. The correlation coefficients between phenotypic traits of lettuces are shown in Table 3, with most traits showing extremely significant positive correlations between each other (*P* < 0.01), but the correlation coefficients between geometric and color traits were less than 0.5, indicating that the geometric and color traits of lettuce differed in how they described the growth and appearance of lettuce. In addition, there were some traits that were highly correlated, indicating that the descriptive properties of these traits were very similar and only reflected slight differences. Among them, the correlation coefficients between the six traits characterized by length (cm) and area (cm^2^) (i.e., PA, PP, CA, CP, AXL, and AXS) exceeded 0.72 (among these, the correlation coefficients between CA and CP reached 0.971). From the perspective of feature engineering, these traits indeed revealed the complex geometrical properties from different perspectives and may partially reflect the morphological and structural differences among lettuce varieties. The correlation coefficients between the three channels of RGB image were more than 0.88, and the correlation coefficient between the *V*-value representing image brightness and the RGB channel was also more than 0.89, indicating that these color indexes might be affected by light intensity. The H channel representing hue showed a significant negative correlation with other color traits to different degrees, which might indicate that value increases of other color channels could lead to decreased hue values within a certain range. ExG is a common color index used to characterize green plants. The effect of green (G) was enhanced by combining RGB channels, and the correlation coefficient with brightness (V) was slightly reduced to 0.85.

**TABLE 3:** Correlation coefficients between static traits of lettuce.

	**PA**	**PP**	**CA**	**CP**	**AXL**	**AXS**	**PAR**	**CAR**	**R**	**G**	**B**	**H**	**S**	**V**	**ExG**
**PA**	1														
**PP**	0.743**	1													
**CA**	0.897**	0.918**	1												
**CP**	0.857**	0.920**	0.971**	1											
**AXL**	0.760**	0.844**	0.873**	0.930**	1										
**AXS**	0.841**	0.855**	0.925**	0.913**	0.724**	1									
**PAR**	0.337**	−0.159**	0.004	−0.093**	−0.227**	0.096**	1								
**CAR**	0.229**	−0.348**	−0.169**	−0.213**	−0.220**	−0.140**	0.849**	1							
**R**	0.095**	0.032	0.054*	0.072**	0.068**	0.061**	0.114**	0.154**	1						
**G**	0.206**	0.097**	0.142**	0.165**	0.150**	0.154**	0.171**	0.212**	0.965**	1					
**B**	0.103**	0.124**	0.112**	0.146**	0.160**	0.107**	–0.006	0.031	0.884**	0.888**	1				
**H**	0.217**	0.234**	0.230**	0.276**	0.259**	0.258**	0.042	0.035	−0.425**	−0.229**	−0.125**	1			
**S**	0.246**	–0.006	0.099**	0.108**	0.055*	0.148**	0.407**	0.445**	0.344**	0.402**	–0.023	−0.145**	1		
**V**	0.199**	0.095**	0.139**	0.162**	0.147**	0.150**	0.165**	0.205**	0.972**	0.999**	0.891**	−0.251**	0.397**	1	
**ExG**	0.082**	–0.003	0.019	0.022	0.011	0.032	0.141**	0.155**	0.855**	0.855**	0.755**	−0.334**	0.273**	0.853**	1

**Correlation is significant at the 0.01 level (two-tailed); *Correlation is significant at the 0.05 level (two-tailed).

**FIGURE 6:**
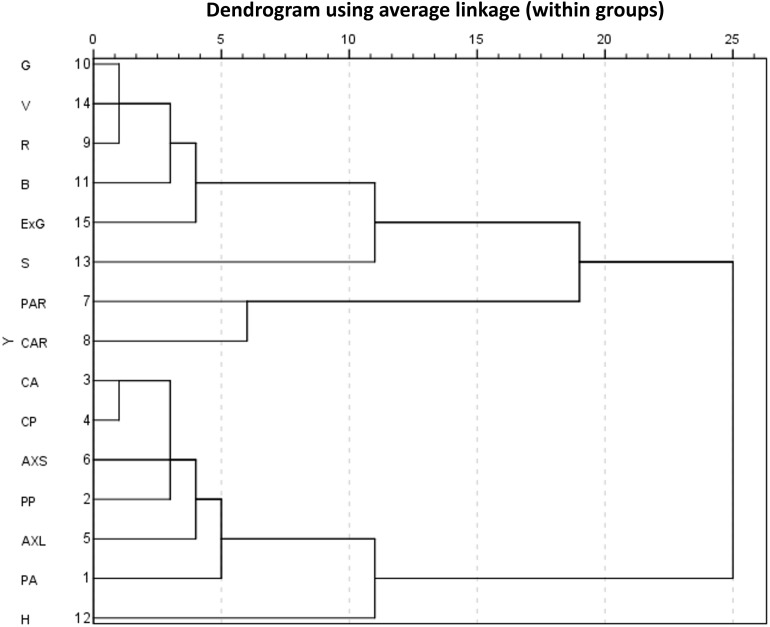
Hierarchical cluster analysis of static traits in lettuce.

Further, hierarchical cluster analysis was conducted on the static traits of lettuce plants. The within-groups linkage cluster method was used to calculate the squared Euclidean distance of static traits measured from the 2036 lettuce images. When the Euclidean distance was 7, static traits could be divided into five categories, as shown in Figure 6. Therein, two color traits, i.e., S and H, were clustered into two individual categories, while the other five color traits were closely related and clustered into one category. For geometric traits, PAR and CAR were dimensionless values representing area proportions of lettuce plants. Other geometric traits with physical dimensions could be classified into the same class. Once the Euclidean distance was 19, all traits were clustered into two categories, but the meaning of each category is not clear or explanatory. Thus, we subsequently performed a principal component analysis (PCA).

Static traits of lettuce plants were standardized and then used for principal component analysis. Eigenvalues greater than 1 were used as a standard for extraction of principal components, and the results are shown in Table 4. The eigenvalues of the first three principal components were greater than 2.22, and the cumulative contribution rate was 84.099%. This indicates the three principal components adequately reflect the basic features of 15 traits. The cumulative contribution rate of the first six principal components reached 96.837%, indicating that the six principal components sufficiently represent all static traits. The contribution rate of the first principal component was the largest, at 38.168%. All the feature vectors representing the length and area were positive and above 0.81, indicating that the geometric structure was still the most direct apparent representation of lettuce, that is, the projected area and size of lettuce plants could best reflect the growth patterns of lettuce plants. The contribution rate of the second principal component was 31.076%, and color traits dominated large feature vector values, among which R, G, V, and ExG feature vectors were all positive and higher than 0.79, indicating that color most differentiated the lettuce plants according to their traits.

**TABLE 4:** Principal component analysis of lettuce traits.

**Phenotypic traits**	**Component**					
	**1**	**2**	**3**	**4**	**5**	**6**
**PA**	0.840	–0.281	0.382	–0.013	–0.169	–0.094
**PP**	0.821	–0.462	–0.149	–0.052	–0.043	0.092
**CA**	0.885	–0.430	0.040	–0.066	–0.087	0.025
**CP**	0.895	–0.428	–0.029	–0.038	0.025	–0.044
**AXL**	0.818	–0.406	–0.125	–0.017	0.092	–0.348
**AXS**	0.844	–0.387	0.107	–0.041	–0.068	0.286
**PAR**	0.075	0.270	0.886	0.118	–0.258	0.163
**CAR**	–0.033	0.377	0.861	0.156	–0.100	–0.248
**R**	0.479	0.850	–0.171	–0.050	–0.015	–0.016
**G**	0.568	0.803	–0.080	0.076	0.103	0.004
**B**	0.499	0.695	–0.334	0.350	–0.096	–0.039
**H**	0.115	–0.469	0.191	0.736	0.422	0.085
**S**	0.269	0.341	0.541	–0.475	0.537	0.047
**V**	0.565	0.807	–0.091	0.061	0.089	–0.001
**ExG**	0.409	0.796	–0.137	0.014	–0.045	0.111
**Eigenvalue**	5.725	4.661	2.228	0.951	0.625	0.334
**Variance contribution rate (%)**	38.168	31.076	14.855	6.340	4.168	2.229
**Cumulative contribution rate (%)**	38.168	69.244	84.099	90.439	94.607	96.837

*Extraction Method: principal component analysis. a. 6 components extracted.*

### Evaluating Dynamic Traits

The growth rate and change in color of lettuce directly reflects the growth and appearance of each plant, which is important in the evaluation and identification of lettuce and of great significance to both breeding and production efforts. In this paper, eight image datasets were collected at 4–5 day intervals during the vegetative growth period (34 days). Based on the image sequence of lettuce plants at each growth point, 15 static characteristics of each plant of each variety were extracted. Then, the variation curves of these static traits (over days) were synthesized in accordance with the time order. Three kinds of lettuce plants with different growth rates and different colors were used to evaluate the growth and accumulation rates of lettuce plants.

**FIGURE 7:**
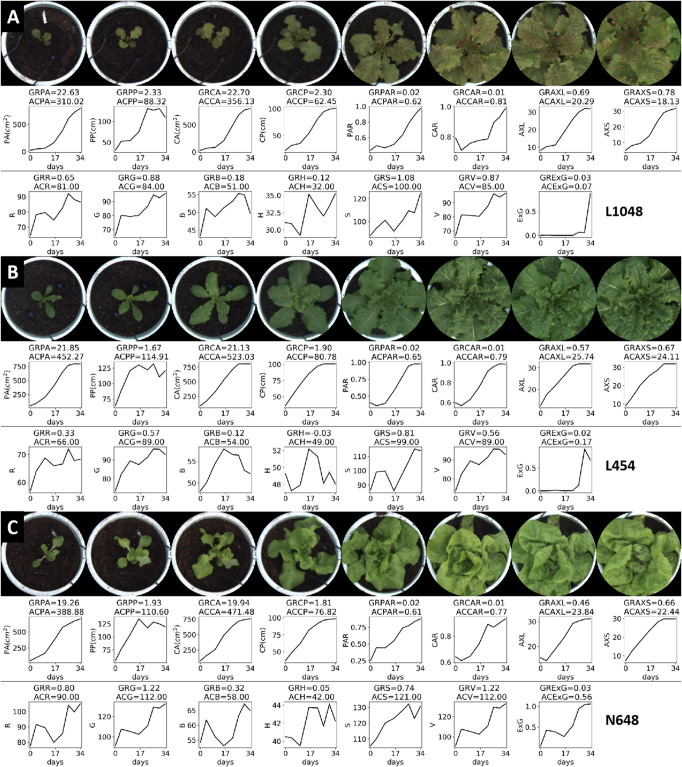
Top-view image sequence and dynamic traits of three lettuce varieties. For the three varieties, 15 static traits were collected from each plant, with corresponding line charts showing the changes in these traits over a 34-day period, and dynamic traits (GR-, AC-) were used to quantify these changes.

Figure 7 shows the top-view image sequence of three lettuce varieties, and the changing curves of the 15 static traits in a 34-day period. We used growth and accumulation rates to quantify these curves. Tables 5, 6 list the dynamic traits of three lettuce varieties according to geometry and color, respectively. Non-parametric tests were performed on the three lettuce samples to evaluate the sample differences. The mean ranks of the geometric traits of varieties L1048, L454, and N648 significantly differed and were 2.06, 2.38, and 1.56, respectively (*P* = 0.046, χ^2^ = 6.143). The mean ranks of the color trait also significantly differed and were 1.82, 1.50, and 2.68, respectively (*P* = 0.005, χ^2^ = 10.582). The chi-square coefficient of the color traits was larger than that of the geometric traits, indicating that color was more differentiated than geometry among the lettuce varieties. Non-parametric tests were also conducted on the geometric and color traits to evaluate the trait differences. The mean ranks obtained are also shown in Tables 5, 6, which show that the ranks of the accumulation rates of lettuce were greater than those of the growth rates, that is, the accumulation rate of lettuce was more strongly differentiated, with ACPP rank being the most differentiated.

**TABLE 5:** Geometry-related dynamic traits of lettuce plants.

	**PA (cm2)**		**PP (cm)**		**CA (cm2)**		**CP (cm)**		**PAR**		**CAR**		**AXL (cm)**		**AXS (cm)**	
	
	**GR-**	**AC-**	**GR-**	**AC-**	**GR-**	**AC-**	**GR-**	**AC-**	**GR-**	**AC-**	**GR-**	**AC-**	**GR-**	**AC-**	**GR-**	**AC-**
**L1048**	22.63	310.02	2.33	88.32	22.70	356.13	2.30	62.45	0.02	0.62	0.01	0.81	0.69	20.29	22.63	310.02
**L454**	21.85	452.27	1.67	114.91	21.13	523.03	1.90	80.78	0.02	0.65	0.01	0.79	0.57	25.74	21.85	452.27
**N648**	19.26	388.88	1.93	110.60	19.94	471.48	1.81	76.82	0.02	0.61	0.01	0.77	0.46	23.84	19.26	388.88
**Ranks**	18	29	15.67	26.67	18.33	30	15.33	23.33	2.33	8.67	1.33	11.67	10.33	19.33	18	29

**TABLE 6:** Color-related dynamic traits of lettuce plants.

	**R**		**G**		**B**		**H**		**S**		**V**		**ExG**	
	
	**GR-**	**AC-**	**GR-**	**AC-**	**GR-**	**AC-**	**GR-**	**AC-**	**GR-**	**AC-**	**GR-**	**AC-**	**GR-**	**AC-**
**L1048**	0.65	81	0.88	84	0.18	51	0.12	32	1.08	100	0.87	85	0.03	0.07
**L454**	0.33	66	0.57	89	0.12	54	–0.03	49	0.81	99	0.56	89	0.02	0.17
**N648**	0.80	90	1.22	112	0.32	58	0.05	42	0.74	121	1.22	112	0.03	0.56
**Ranks**	9	23.67	12	25.33	5.33	22	3.33	21	12.67	27.67	11	26.33	3.33	5.67

The projected area (PA) represents the coverage or spreading area of the lettuce from the top-view, and it is the most commonly used trait to characterize digital biomass. The growth rates of L1048, L454, and N648 varieties were observed to gradually increase, but the accumulation rates differed in the way they changed. In the lettuce seedling stage, the growth rate of L1048 was slow, but accelerated after 17 days, reaching the maximum GRPA at 34 days, and because of the lag in its accelerated growth time, its ACPA was lowest among the three varieties. Similar trends were also found among geometry-related traits, including PP, CA, CP, AXL, and AXS. Moreover, the dynamic PP and CP that described the contour of the lettuce provide richer information, not only reflecting differences in the projected area of the lettuce, but also representing the complexity of lettuce boundaries (i.e., leaf margins) to some extent. On the whole, all the growth rates related to area and size for L2018 were the highest, while its accumulation rate was the lowest, which might indicate that growth rate was sensitive to the initial state, while the accumulation rate was not. This pattern revealed that the time point at which the varieties started accelerated growth, was negatively associated with the accumulation rate. For L454, the rapid growth of lettuce leaves in the pot region maximized the accumulation rate of all traits related to area and size, indicating that the accumulation rate more accurately reflected the growth status of plants in each pot region. Generally, PAR and CAR described the degree to which the plant canopy filled the pot region, and they gradually increased with time.

The color-related dynamic traits of three lettuce varieties are listed in Table 6. The cumulative rate of color change reflected the average color characteristics of the plants throughout the growth period, but the R, G, and B channels of the plants need to be combined to reflect their true colors. For the lettuce varieties L1048 and L454, which have no obvious visual differences, their ACG, ACB, ACS, and ACV values were quite close, indicating that the color differences in G, B, S, and V channels were slight. ACH better described the difference between green and non-green plants. For the dark green L454 and light green N648 varieties, ACG and ACR became effective green difference descriptors. In the comparison of the three colors of lettuce, ACExG always exhibited a strong color differentiation, so the introduction of more color or vegetation indices, such as NGRDI (Hunt et al., 2005), RGRI (Gamon and Surfus, 1999), NDVI (Rouse et al., 1974), DVI (Jordan, 1969), etc., might enhance the color representation of lettuce varieties with different and rich colors.

### Verification of Lettuce Traits

The *in situ* measurement and evaluation of lettuce traits is labor-intensive and highly subjective, especially for geometric and color trait measurement. More importantly, a large number of traits with variable characteristics are difficult to measure manually. In this work, a series of geometric and color traits of lettuce were extracted from top-view images. To verify the measurement accuracy of the phenotype platform, 497 lettuce plants were collected for an individual trial for artificial geometrical measurement and color grading. The canopy width (CW) of lettuce was defined as the longest distance of leaf expansion, which was selected as the manual measurement index owing to its simplicity and consistency. Moreover, the main color of lettuce plants in this particular experiment green, though some purple and red-purple coloration also occurred. Limited by the human eye’s color resolution, we roughly divided the colors of lettuce into light green, dark green, and purple categories.

**FIGURE 8:**
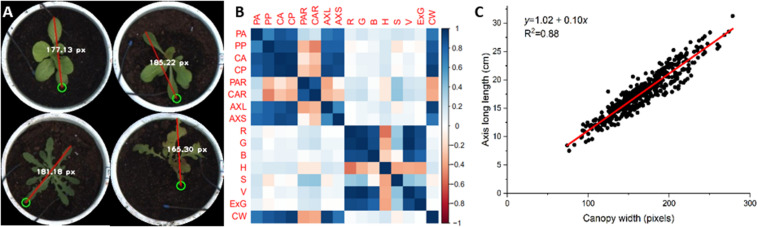
Canopy width (CW) as assessed by interactive measurement and correlation analysis with static traits of lettuce plants. **(A)** CW as assessed by interactive measurement. **(B)** Correlation between canopy width and static traits of lettuce. **(C)** Linear fit of canopy width and long axis length (AXL).

Based on collected lettuce images, we interactively measured the canopy width of each lettuce plant from the pot image, as shown in Figure 8A. Correlation analysis between canopy width and all static traits of lettuce showed correlation coefficients between CW and color traits of less than 0.18 (Figure 8B). For geometric traits, CW was negatively correlated with PAR (−0.41) and CAR (−0.38), but there were positive correlation coefficients with the other geometric traits (more than 0.72). The two highest correlation coefficients were those for CW with CP and AXL (both about 0.94). As the definition and measurement of CW are highly consistent with AXL, we calculated the coefficient of determination (*R*^2^) between CW and AXL, as shown in Figure 8C. Both manual *in situ* and interactive measurements depend on the experience of the experimenter, thus leading to subjective results. Comparatively speaking, image-based phenotyping can provide more objective, stable, and consistent geometric measurements. Additionally, many important geometric traits, such as projected area (CA) and canopy contour, are almost impossible to measure manually.

**FIGURE 9:**
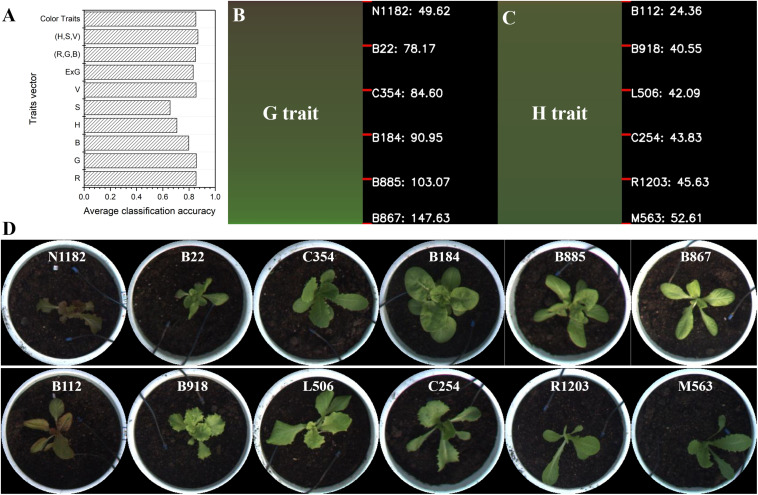
Color grading of lettuce varieties. **(A)** The average classification accuracy of 10 kinds of feature vector combinations based on the kNN algorithm. **(B)** Color grading chart of lettuce varieties based on the color trait G. **(C)** Color grading chart of lettuce varieties based on the color trait H. **(D)** Sorted lettuce varieties with different color grades quantified by G and H, respectively.

Lettuce color, which is regulated by multiple loci, shows dynamic variation during lettuce growth and development, which makes its genetic analysis extremely complex (Su et al., 2020). The accurate quantification and classification of lettuce color is important in studying the genetic and molecular mechanism of leaf color and its components (i.e., anthocyanins) for lettuce breeders. In general, lettuce color can only be roughly classified by visual observation, such as into light green, dark green, red, and other color types. Thus, it is hard to accurately describe and quantify color variation for many lettuce varieties. In this work, the color of 497 lettuce varieties was investigated and graded manually into three categories: light green (0), dark green (1), and purple (2). For all lettuce samples, an artificial color grading index was used as the category, and color traits of lettuce were used to generate the feature vector. Then, the k-nearest neighbors (kNN) algorithm was utilized to calculate the average classification accuracy, which indicated the consistency between the color traits extracted from the image and artificial color grading indices. For the seven color traits (Table 1), we constructed 10 different feature vector combinations for kNN training, which were R, G, B, H, S, V, ExG, (R,G,B), (H,S,V), and color traits (comprising seven traits). In each kNN train process, lettuce samples were first divided into four groups, and each group was further divided into training and test sets. The kNN model was trained based on each feature vector combination, and the average classification accuracy of the kNN classifier was calculated based on four-fold cross-validation. Finally, the average classification accuracy of 10 kNN models were collected and the results are shown in Figure 9A. Among the 10 feature vector combinations, the combination of H, S, and V reached the highest classification accuracy of 86.78%, and color traits (a combination of the seven color traits) had a slightly lower classification accuracy of 85.18%. For feature vectors that only contained one color trait, G had the highest classification accuracy of 85.57%, and S had the lowest classification accuracy of 65.52%. Notably, the classification accuracy of H was only 70.75%. The above results indicate that the color traits have a high consistency with artificial color grading indices, and the classification error of kNN is likely owing to the subjectivity and uncertainty of artificial color grading.

We further calculated the value range of each color trait for all lettuce varieties and then sorted the lettuce varieties according to each trait value to obtain a color grading chart (CGC) for the assessed lettuce varieties. CGC defines a vertical color bar (VCB) that indicates color trait variation of the sorted lettuce varieties; thus, each height position in a VCB represents the corresponding lettuce variety and its trait value. Figures 9B,C show the corresponding color grading charts (CGCs) based on G and H respectively. We divided each VCB into five equal height intervals and marked the representative lettuce varieties and trait values at each interval point. The value ranges of different color traits were quite different, e.g., the value range of G was from 49.62 to 147.63, which was significantly greater than that of H from 24.36 to 52.61. Based on the CGC of G and H, the images of representative lettuces with different color grading indices are shown in Figure 9D. Although lettuce varieties were differentiated according to color grading based on G and H, the continuous trend in lettuce color was visually obvious. Artificial color grading indices (light green, dark green, and red) could also be visually observed between certain small intervals in CVB, but color identification in other intervals was ambiguous for many lettuce varieties. Image-based color grading provided a more consistent and reliable basis for color quantification, and the CGC graphs were helpful for identify trait differences among all varieties.

Compared with manual investigation, morphological measurement and color grading based on the present phenotyping platform improved the efficiency and accuracy of the phenotype investigation for large-scale lettuce cultivation. More importantly, this method can be used to automatically measure multiple time points of lettuce cultivation, so as to assess dynamic variation among all kinds of traits. Thus, this research lays a foundation for identifying and mapping genes related to geometry and color from lettuce germplasms.

## Discussion

In this plant high-throughput phenotyping study, stable and high-frame-rate image acquisition by industrial cameras was the most efficient and economical method for phenotyping and analysis. To describe the dynamic changes in traits for large numbers of plants during the growth period, thousands of images collected in batches need to be processed; thus, automated and efficient image processing and trait extraction is a great challenge. In this research, a high-throughput greenhouse-based phenotyping platform was used to collect top-view images during the vegetative growth period for hundreds of lettuce varieties, and an image analysis method was developed to automate the extraction of static and dynamic traits of these lettuce varieties.

We designed multistage CNN models to perform object detection and semantic segmentation of lettuce plants and then made a pipeline to process the image sequence into plant phenotypes. This scheme could improve the robustness and flexibility of lettuce phenotyping systems. An efficient pot detection model was trained to locate plants from the image sequence, with a detection accuracy reaching 99.8% and a measurement error of pot size of less than 3%. The pots detected from the image sequence were matched to the corresponding plants. A high-precision semantic segmentation model was also used to analyze the growth sequence of each lettuce plant, which accurately segmented lettuce of different varieties throughout their vegetative growth stages. The F1 scores in the training, verification, and test sets were each over 97%. The high detection and segmentation accuracy may be related to the relatively simple greenhouse setting and the relatively low background noise in the annotated dataset.

Data collected at eight time points were used to investigate dynamic traits, including the growth and accumulation rates of lettuce varieties. Mean rank of the accumulation rate was higher than one of the corresponding growth rate and thus more appropriately represented trends in dynamic variation of various static traits. Notably, the present study only calculated the various traits of lettuce plants within the perimeter of each pot, and because of overlap among adjacent lettuce plants at later growth stages, it was difficult to completely separate each individual lettuce from the later top-view images. Therefore, we limited the “phenotyping region” to the area within each pot for two reasons. First, the pot region provided a standard threshold for assessing the time at which lettuce plants grew to the boundaries of pots. Thus, the data before this growth point could be used to quantify growth rates of lettuce plants, while the data collected after this growth point could still be used to quantify other dynamic traits (such as color changes etc.) over longer growth time periods. Second, the fixed pot region is helpful in evaluating dynamic growth of lettuce plants under the same criteria, and it reduces ambiguities in the automated phenotyping analysis pipeline. From the perspective of methodology, the “phenotyping region” can also be redefined according to the specific purposes of applications. To enable more accurate monitoring of lettuce growth, it is better to reduce the flowerpot density to eliminate interactions between adjacent plants. However, this inevitably decreases the detection flux and efficiency.

A large number of traits can be extracted from plant images, and various novel indicators can also be determined from different dimensions. However, the interpretability of traits still needs to be explored in depth, specifically in terms of how these traits describe the detailed static and dynamic features of plants. In this work, we quantified the static and dynamic traits (i.e., growth and accumulation) from top-view images of lettuce plants. To eliminate overlap between adjacent plants at the later stage of vegetative growth of lettuce and unify the standards for dynamic phenotyping and quantification, the growth status of lettuce was continuously and stably detected and evaluated only within the circular regions of pots. Fifteen static traits were used to describe the geometry and color status of each lettuce at specific growth points. The correlation, cluster, and principal component analyses of the 2036 lettuce plants showed that the geometry and color traits of lettuce differed, and their correlation coefficients with each other were less than 0.5. Meanwhile, within the same types of traits, there were some pairs of traits with extremely high correlation coefficients. It is necessary to combine these similar traits to describe much less obvious features of plants.

To verify the reliability of this phenotyping platform, we evaluated the geometric and color traits of 497 lettuce varieties, respectively. Canopy width (CW) of lettuce plants was chosen as a geometric indicator for manual human measurements, and correlation analysis results showed a significant correlation between CW and PA, PP, CA, CP, AXL, and AXS traits. The coefficient of determination (*R*^2^) between CW and AXL was 0.88. In addition, we performed artificial color grading and classified all lettuce varieties into three categories: light green, dark green, and red. The kNN algorithm was used to calculate the average classification accuracy, and the feature vector combination of H, S, and V reached the highest classification accuracy of 86.78%. Further, the color grading charts (CGC) of lettuce varieties demonstrated more stable and consistent color quantification and classification abilities. The above results demonstrate the reliability of the phenotyping platform in measuring geometric traits and grading color differences for large-scale lettuce cultivation.

Image-based high-throughput phenotyping can be more objective and reliable, especially for continuous traits, and these static and dynamic traits provide a basis for further refining the design of trait indicators of lettuce based on images, and more geometric and color traits could be designed from different perspectives to improve the ability of the system for phenotyping plants. Moreover, the presented method also has reference values for other optical imaging technologies, such as multispectral and thermal infrared imaging. Multiple sensors can be integrated to investigate lettuce traits across more dimensions, especially for physiological and water stress traits of lettuce plants. These technologies have substantial potential to discover new traits that breeders cannot assess via traditional methods, and the resulting static and dynamic traits could be employed in genome-wide association studies (GWAS) of these lettuce varieties.

## Conclusion

In this paper we proposed an automated phenotyping pipeline for the non-destructive, high-throughput detection and phenotyping of lettuce varieties in a greenhouse environment. We developed multistage CNN models for pot detection and plant segmentation. The pot detection and semantic segmentation models achieved satisfactory analysis results in terms of efficiency and accuracy, which was used to integrate image sequence capture with plant phenotyping in a single pipeline. We have investigated the static and dynamic traits of lettuce plants and interpreted the relationship between static and dynamic traits, using both geometry- and color-related traits. These traits were useful descriptors of the digital biomass and quantity of these lettuce varieties. To the best of our knowledge, this is the first work to study the accumulation rates of static straits, which more accurately reflect the dynamic growth of plants. Finally, we evaluated and validated the application of high-throughput phenotyping platforms in geometry measurement and color grading of large-scale lettuce cultivation. The proposed method is not only suitable for vegetables in greenhouses, but could also be extended to crops such as maize, wheat, and soybean.

## Data Availability Statement

All datasets generated for this study are included in the article/supplementary material.

## Author Contributions

JD, XY, and XG conceived and designed the experiments. JF, XL, and YQ carried out greenhouse management. JD designed the high-throughput phenotyping platform, developed the lettuce phenotyping pipeline, and performed data collection, processing and analysis. JD drafted the manuscript. XY and JD discussed the analysis results and revised the manuscript. All authors contributed to the article and approved the submitted version.

## Conflict of Interest

The authors declare that the research was conducted in the absence of any commercial or financial relationships that could be construed as a potential conflict of interest.
